# From first infection to reinfection: Comparing Nucleocapsid antibody kinetics in vaccinated and unvaccinated adults

**DOI:** 10.1016/j.vaccine.2025.127593

**Published:** 2025-08-13

**Authors:** Kathleen M. Lindsey, Zachary Farrell, Rebecca Tutino, Theresa Kowalski-Dobson, Zijin Chu, Carmen Gherasim, Shuwei Cai, Gabriel Simjanovski, David Manthei, Emily Stoneman, Florian Krammer, Riccardo Valdez, Aubree Gordon

**Affiliations:** a Department of Epidemiology, School of Public Health, University of Michigan, Ann Arbor, United States; b Department of Pathology, Michigan Medicine, University of Michigan, Ann Arbor, MI, United States; c Division of Infectious Diseases, Michigan Medicine, University of Michigan, Ann Arbor, MI, United States; d Department of Microbiology and Center for Vaccine Research, Pandemic Preparedness (C-VaRPP), Department of Pathology, Molecular and Cell-Based Medicine, Icahn School of Medicine at Mount Sinai, New York, NY, United States, and Ignaz Semmelweis Institute, Interuniversity Institute for Infection Research, Medical University of Vienna, Vienna, Austria

**Keywords:** SARS-Cov-2, Epidemiology, Serology, Nucleocapsid, Vaccination

## Abstract

**Objectives:**

Determining infection histories of individuals with prior immunity is critical to studying SARS-CoV-2 imprinting and vaccine responses. To better understand the response to infection, we examine nucleocapsid protein antibody dynamics around first and second SARS-CoV-2 infections in vaccinated and unvaccinated adults.

**Methods:**

The presence and levels of nucleocapsid-antibodies were measured around first and second SARS-CoV-2 infections. Antibody waning was measured with accelerated failure time models.

**Results:**

After first-infections 95.8 % (88.5–98.6 %) of unvaccinated and 89.4 % (86.5–91.7 %) of vaccinated participants seroconverted. Vaccinated adults had 45.5 % shorter seropositivity (event time ratio = 0.55, 95 %CI: 0.45–0.66). After second-infections, 89.1 % (76.9–95.3 %) of vaccinated and 94.4 % (74.2–99.0 %) of unvaccinated participants had a 4-fold rise in antibody levels. Regardless of vaccination, antibody levels after second-infections are significantly higher than after first-infections.

**Conclusions:**

These findings suggest nucleocapsid serology can inform researchers about past infections regardless of exposure status, and potentially differentiate between initial infections and reinfections.

## 1. Introduction

Determining infection histories of individuals with prior immunity is critical for studying SARS-CoV-2. However, high levels of binding spike-antibodies from infections and vaccinations limit their rise upon subsequent exposures [1]. Relying on spike serology alone may reduce the ability to detect infections in vaccinated individuals and lead to overestimations of vaccine effectiveness. This issue has been observed in influenza vaccine trials and is suspected in SARS-CoV-2 vaccine trials [2,3].

To mitigate this bias, current methods for identifying past infections focus on using serology to detect nucleocapsid proteins (NP) [4]. This approach is effective because many vaccines, especially those manufactured in the US, target a full-length spike protein [5,6]. Since these vaccines do not target other structural proteins, immune response to the highly conserved nucleocapsid protein is a reliable indicator of past infections [7].

Although the presence of NP-antibodies is an accepted method to indicate past SARS-CoV-2 infection, uncertainty remains regarding its efficacy in detecting reinfections and variations by vaccination status [8]. Herein, we analyze NP-antibody dynamics considering individual vaccination status and prior infections to better understand the NP-antibody response to infection in vaccinated and unvaccinated individuals.

## 2. Methods

### 2.1. Participants

Participants, aged ≥18 years, were sampled from the Immunity Associated with SARS-CoV-2 (IASO) study, a prospective longitudinal cohort study in Ann Arbor, Michigan [9], and the Household Influenza Cohort Study (HICS), an ongoing prospective household cohort study in Managua, Nicaragua [10]. In both studies, participants were monitored for SARS-CoV-2 infection with periodic blood samples collected regardless of infection status. A subset of participants underwent timed blood draws following infection. In HICS, all participants SARS-CoV-2 infections were confirmed with RT-PCR. In IASO, most participant infections were RT-PCR confirmed, others were confirmed through testing, including rapid antigen tests or changes in anti-spike antibody levels following a reported positive from a participant. Vaccination status was collected through participant self-report and confirmed by documentation (i.e. vaccine card, medical record, vaccine registry) when possible. HICS participants were included in this analysis to contribute to the group of participants with unvaccinated first infections due to the high rate of vaccination in the IASO cohort. IASO participants with paired samples and documented infections were subset into groups by vaccination status and first or second infection. These groups were selected either by a random sample or the entire group was selected based on sample size.

### 2.2. Ethics statement

Studies were approved by the institutional review boards at the Nicaraguan Ministry of Health and the University of Michigan Medical School and conducted in accordance with the Helsinki Declaration of the World Medical Association. All participants gave written informed consent.

### 2.3. Laboratory testing

Serum samples were tested for NP-antibodies with enzyme-linked immunosorbent assays (ELISA) developed by the Krammer Laboratory and modified for NP-antibodies detection [11]. ELISAs were performed on paired serum samples taken around infection. Seroconversion was assessed using a single dilution ELISA. For IASO samples, additional ELISAs were performed with the adapted Krammer protocol to quantify anti-NP SARS-CoV-2 immunoglobulin-G (IgG) antibody titers.

### 2.4. Statistical analysis

Participants’ paired samples were taken pre-infection, day of first positive molecular or antigen test, and 14–90 days post-infection unless otherwise specified. To observe seroconversions after first infection, we compare paired samples in those with negative pre-infection samples. Categorical data were compared with *χ*^*2*^ test, continuous measures were analyzed with Kruskal-Wallis tests, and trends with Cochran–Armitage. Mild symptom presentation was defined as infections that did not report respiratory discomfort or require medical attention.

Duration of NP-antibody seropositivity was determined with a random selection of 100 vaccinated and 50 unvaccinated IASO participants with apparent first infections. First infections were defined as those in participants without prior SARS-CoV-2 infection history. This includes infections with observed seroconversion and, when pre-infection samples were unavailable, infections in 2020 or 2021 before widespread variant circulation in the study. Participants had ≥4 samples post-infection taken <6 months apart, with the first sample taken 14–90 days post-infection. Accelerated failure time (AFT) models with Weibull distributions and right censoring compare the duration of NP-seropositivity after first infection by vaccination status with event time ratios (ETR) as performed elsewhere [12].

To observe changes in NP-serology around a reinfection, we compare antibody levels in paired samples around a confirmed second infection. Here, pre-infection samples were restricted to 1–90 days pre-infection. Antibody levels after first and second infections were compared to observe potential boosting after multiple exposures. Analyses were performed in SAS (9.4) and R (V.4.4.1).

## 3. Results

This analysis includes samples from 739 SARS-CoV-2 infected individuals: 700 first and 65 second infections. Among these, 92.3 % of participants are from the IASO cohort and mostly vaccinated at time of infection (Table S1). Most infections were confirmed with RT-PCR and occurred during the Omicron peak in early 2022 (78.3 %; Table S2, Fig. S1).

### 3.1. First SARS-CoV-2 infections

To estimate prevalence of NP-antibody seroconversion following initial SARS-CoV-2 infection, we analyzed paired samples from 555 vaccinated IASO participants and 72 unvaccinated IASO (*n* = 15) and HICS (*n* = 57) participants. The median time since the last vaccine dose among the vaccinated was 174 days (range: 0–618; SD: 106.7).

After initial infection, 90.1 % (565/627; 95 %CI: 87.5–92.2 %) of participants seroconverted. Seroconversion prevalence was similar among participants, with 95.8 % (69/72; 95 %CI: 88.5–98.6 %) of unvaccinated participants seroconverting, compared to 89.4 % (496/555; 95 %CI: 86.5–91.7 %) among vaccinated (*P* = 0.0839), and did not vary by variant (p-trend = 0.5297). While vaccinated participants experienced more mild infections, severity was similar by seroconversion (Table S3).

### 3.2. Duration of NP positivity following first infection

**Fig. 1.**
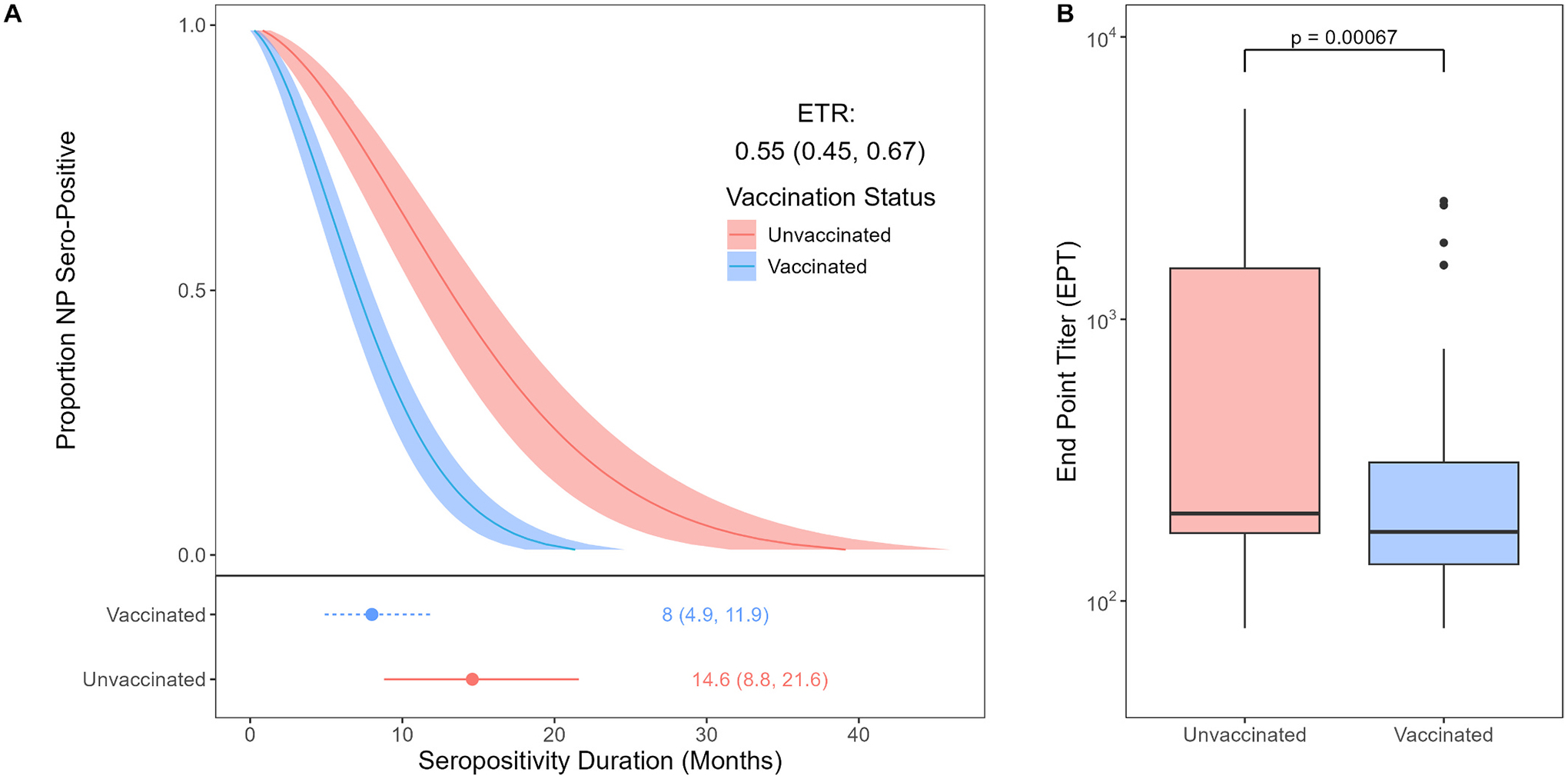
Serology following first infection in 142 IASO participants. A. Duration of NP-antibody seropositivity by vaccination status at infection. AFT model of vaccinated vs unvaccinated infections (top), including ETR and IQR of mean duration with 95 % confidence intervals (bottom). B. Initial NP-antibody level of the first sample after first infection, shown on the log scale. Significance test: Kruskal-Wallis.

Of the 150 randomly selected IASO participants, eight were excluded due to a lack of pre-infection samples, which leads to an inability to determine the duration of seropositivity. On average, participants contributed 7 samples (range: 4–14) over a period of 14.6 months (range: 5–33). Vaccinated individuals exhibited a 45.5 % shorter duration of seropositivity compared to unvaccinated individuals (ETR = 0.55; 95 %CI: 0.45–0.66; Fig. 1A). Participants maintained detectable levels of NP-antibodies for an average of 12.6 months (IQR: 9.9–18.1). Six months after initial infection, 91.3 % (95 %CI: 79.7–96.6 %) of unvaccinated individuals and 83.3 % (95 %CI: 74.6–89.5 %) of vaccinated individuals maintained detectable levels of NP-antibodies (*P* = 0.2013). In 140 participants with available samples, median antibody levels were significantly higher in unvaccinated than vaccinated individuals (*P* = 0.00067; Fig. 1B).

### 3.3. NP antibody dynamics around SARS-CoV-2 reinfections

Using NP-seropositive IASO participants, we compare paired sample titers around a second infection between 18 unvaccinated adults and 46 vaccinated adults. Following second infection, 90.6 % (95 %CI: 81.0–95.6 %) of participants had a ≥ 4-fold rise in NP-antibody titers.

**Fig. 2.**
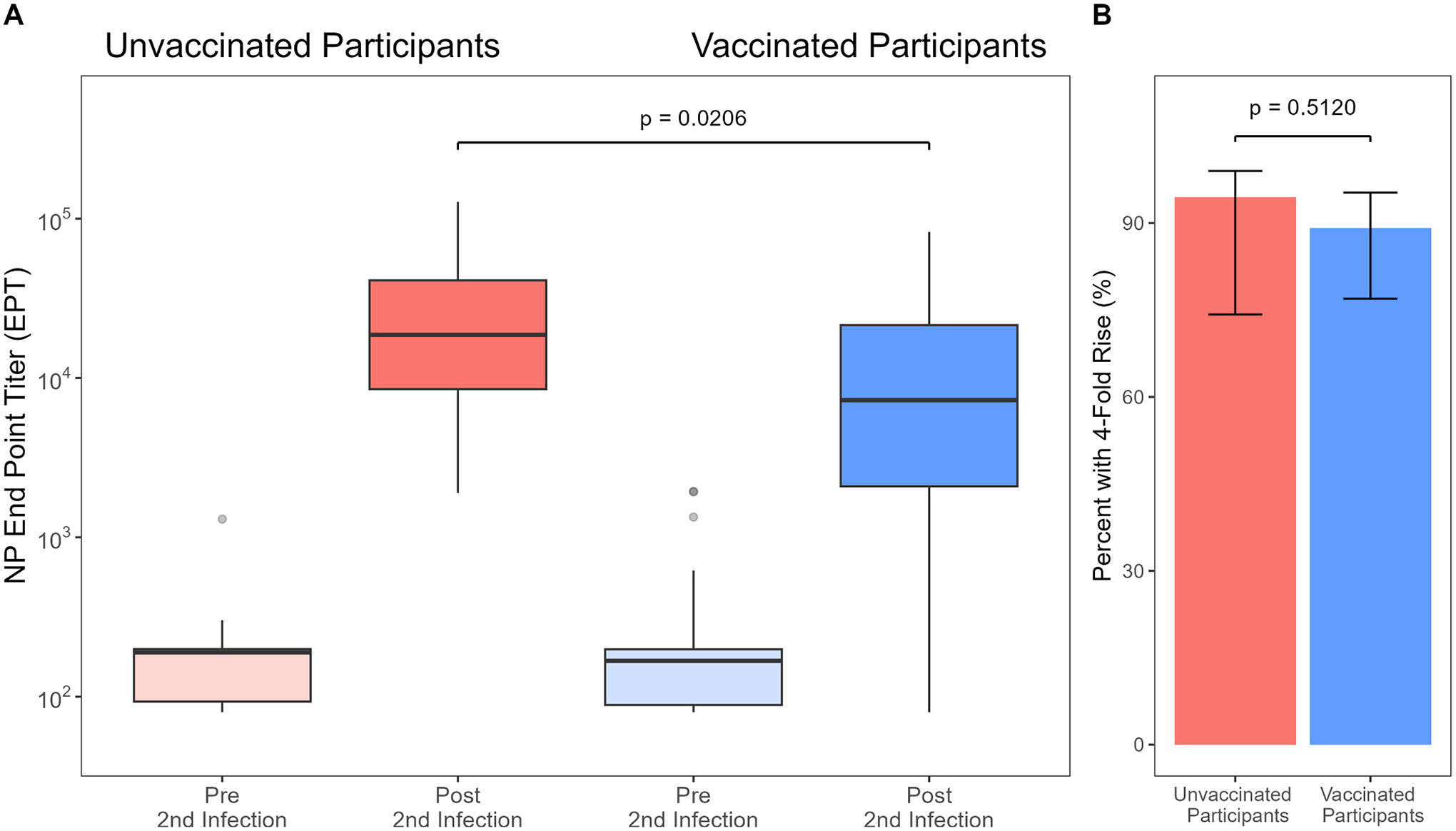
Four-fold rise in antibody levels following a confirmed second infection in vaccinated and unvaccinated IASO participants. A. Distribution of antibody titer (EPT) before and after infection by vaccination status. Pre- and post-infection antibody titers are displayed on the log scale. Black bars indicate the median value. Left, titer changes in second infections in unvaccinated individuals. Right, titer changes in second infections in vaccinated individuals. B. Percent of participants with a 4-fold rise after a second infection. Error bars show 95 % Wilson confidence intervals.

The median fold change in antibody level for those unvaccinated and vaccinated was 111.7 and 35.6 respectively (*P* = 0.0066; Fig. 2A). The prevalence of a 4-fold rise in NP-antibody titers was 89.1 % (95 %CI: 76.9–95.3 %) in vaccinated participants and 94.4 % (95 %CI: 74.2–99.0 %) in unvaccinated participants (*P* = 0.5120; Fig. 2B). Median titers following second infections were 18,660 (range: 1901–127,400) in unvaccinated individuals compared to 7337.5 (range: 80–82,610) in vaccinated individuals (*P* = 0.0206). Infection severity did not significantly differ by vaccination status or by presence of a 4-fold rise (Table S3).

### 3.4. Antibody levels following a first or second infection

**Fig. 3.**
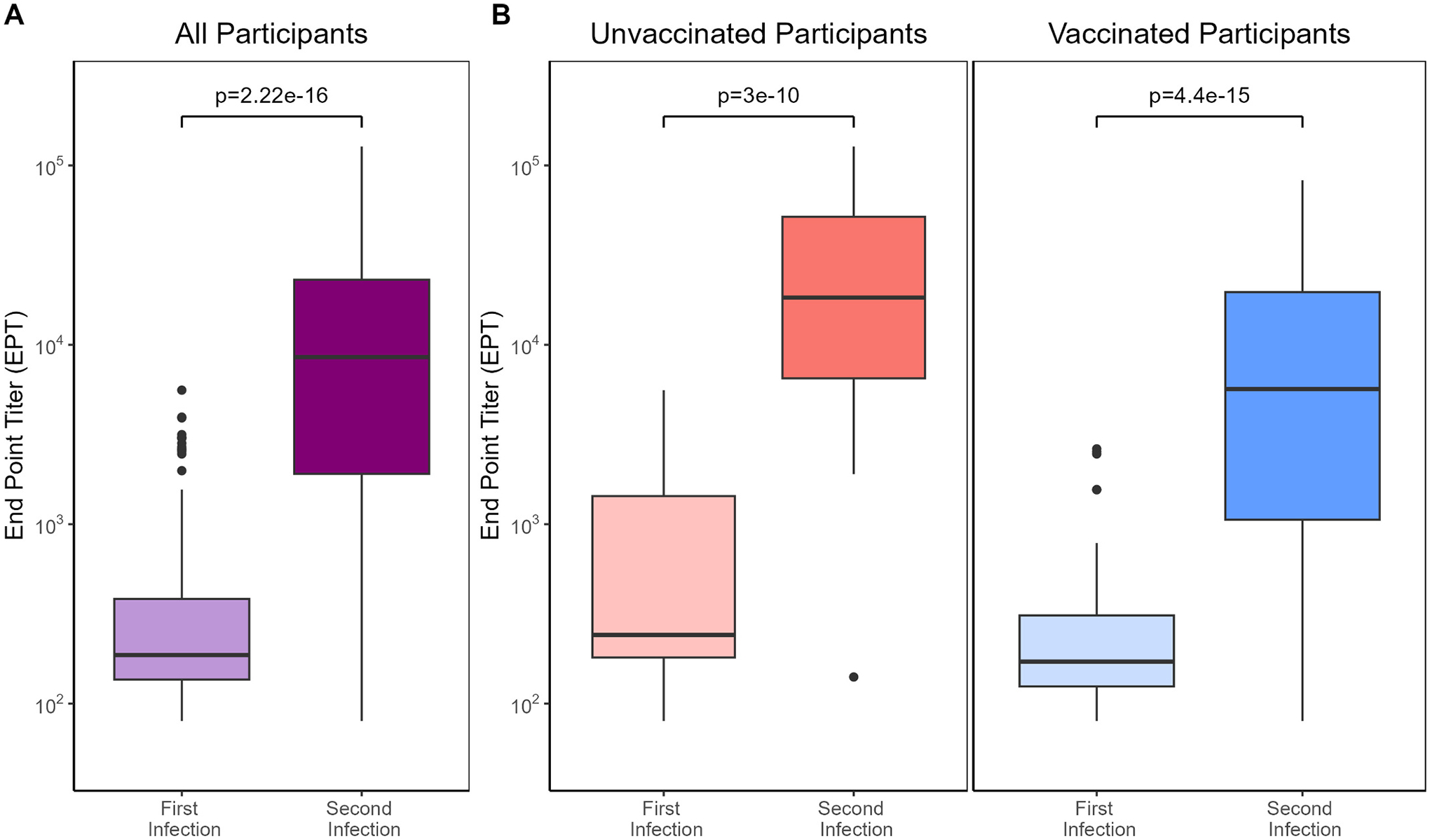
Participant antibody levels reached after a first or second SARS-CoV-2 infection. A. Includes both vaccinated and unvaccinated participants. B. Compares antibody level by participants vaccination status.

Comparing IASO participants’ antibody levels following a primary infection (48 unvaccinated and 100 vaccinated) to those after a second infection (18 unvaccinated and 46 vaccinated), we observed a clear boosting effect (Fig. 3A). Individuals with a first infection reached a mean antibody level of 545.16 compared to 19,870.36 in individuals following a second infection (*P* < 0.0001). Unvaccinated participants reached higher antibody levels than vaccinated participants after a first infection (P < 0.0001), and after a second infection unvaccinated participants still reached higher antibody levels (*P* = 0.0121; Fig. 3B).

## 4. Discussion

Differential ability to detect SARS-CoV-2 infections using serology endpoints between vaccinated and unvaccinated participants can lead to biases like overestimating vaccine effectiveness or the impact of risk factors. Our analysis uses longitudinal sampling to examine nucleocapsid protein serology as a measure to better understand infection histories in the general population. We found clear patterns of NP-antibody seroconversion after first infections and boosting after reinfections, indicating that NP antibody levels may help reconstruct infection histories in both vaccinated and unvaccinated adults. The duration of seropositivity varied by vaccination status, likely due to different magnitudes of NP-antibody rise observed after first infection. Thus, while NP serology is a robust method for infection monitoring, ideally samples are obtained in intervals no longer than 6 months apart.

We found that 90.1 % of individuals seroconvert for antibodies against the nucleocapsid protein after a first infection, regardless of vaccination status. This is well within the range found in other studies [13–15]. While seroconversion proportions were comparable between unvaccinated and vaccinated individuals, those who were vaccinated had a shorter duration of NP seropositivity. This reduced longevity of antibodies in vaccinated participants has been documented [12,15], and was expected, partially due to the effectiveness of SARS-CoV-2 vaccines which limit the number of viral copies and thus limits exposure to the nucleocapsid protein [16]. Other mechanisms may also contribute to this difference in antibody duration, such as class switching toward IgG4 following repeated mRNA vaccination, which has been associated with reduced effector functions and may affect the durability of the antibody response, though its role in nucleocapsid-specific immunity remains unclear and requires further study [17].

Similarly to another study, we observed that antibody levels following a first infection were significantly higher in unvaccinated compared to vaccinated individuals [18], which we contend also contributes to the longer duration of seropositivity. Our finding that seropositivity proportions in vaccinated and unvaccinated individuals were not significantly different 6-months post first infection, supports that testing serum samples collected every 6 months should be adequate to detect infections. However, we recommend conducting sensitivity analyses to assess the impact of differential detection by population.

We observed significantly higher NP-antibody levels after a secondary infection compared to a first infection, regardless of vaccination status. Studies of antibody kinetics after reinfections in a highly vaccinated population have also documented a similar boosting trend [19;20]. Therefore, NP-antibody levels following infection could potentially differentiate between primary and secondary SARS-CoV-2 infections.

This analysis has several limitations. First, it is restricted to first and second infections, and further research is needed to assess whether our findings apply to more complex SARS-CoV-2 infection histories. Second, the high vaccination rate in the IASO cohort reduced the sample size of unvaccinated participants, limiting the precision of our estimates. However, we addressed this by including participants from a second cohort for first infection analyses. Finally, pre-infection samples were unavailable in some scenarios, such as when participants were enrolled at the time of an acute SARS-CoV-2 infection or enrolled shortly after a reported infection. In these cases, if the infection occurred in 2020 or early 2021, prior to widespread variant circulation in the study area when reinfection was extremely rare, and there was no documented or suspected prior infection, it was classified as a first infection. While this approach reduces the likelihood of misclassification, we acknowledge the possibility of undetected infections.

This work characterizes nucleocapsid serology response after first infection and second infections and supports that it may be useful to detect SARS-CoV-2 infections in both unvaccinated and vaccinated individuals. Our findings highlight the differential antibody responses between these groups, emphasizing the need for regular monitoring to accurately capture infection histories. Identifying first infections remains important for understanding immunologic imprinting, which can shape the magnitude and breadth of responses to subsequent infection and vaccinations, and thus has implications for vaccine development, effectiveness evaluations, and disease burden modeling. As we continue to transition into SARS-CoV-2 endemicity, further research is essential to fully understand the long-term dynamics of NP-antibody responses and their utility in detecting infections.

## Supplementary Material

1

## Data Availability

De-identified data needed to create figures, run SAS code, and R code can be made available. However, since data is on human subjects, full data access is restricted to collaborators who have been IRB approved.

## Appendix A. Supplementary data

Supplementary data to this article can be found online at https://doi.org/10.1016/j.vaccine.2025.127593.
