# Supplementary material for: From first infection to reinfection: Comparing Nucleocapsid antibody kinetics in vaccinated and unvaccinated adults
Source: Vaccine. Author manuscript; Available in PMC 2026 Jun 5. (PMC13238270; doi:10.1016/j.vaccine.2025.127593)
Supplement: 1 [file NIHMS2180914-supplement-1.docx]

Supplement for

**From First Infection to Reinfection: Comparing Nucleocapsid Antibody Kinetics in Vaccinated and Unvaccinated Adults**

Kathleen M. Lindsey^1*^, Zachary Farrell^1*^, Rebecca Tutino^1^, Theresa Kowalski-Dobson^1^, Zijin Chu^1^, Carmen Gherasim^2^, Shuwei Cai^1^, Gabriel Simjanovski^1^, David Manthei^2^, Emily Stoneman^3^, Florian Krammer^4,^ Riccardo Valdez^2†^, Aubree Gordon^1†^

Author Affiliations:

1. Department of Epidemiology, School of Public Health, University of Michigan, Ann Arbor, MI, United States
2. Department of Pathology, Michigan Medicine, University of Michigan, Ann Arbor, MI, United States
3. Division of Infectious Diseases, Michigan Medicine, University of Michigan, Ann Arbor, MI, United States
4. Department of Microbiology and Center for Vaccine Research, Pandemic Preparedness (C-VaRPP), Department of Pathology, Molecular and Cell-Based Medicine, Icahn School of Medicine at Mount Sinai, New York, NY, and Ignaz Semmelweis Institute, Interuniversity Institute for Infection Research, Medical University of Vienna, Vienna, Austria

* These authors contributed equally; ^†^ Co-senior authors

Corresponding Author: gordonal@umich.edu

Table of Contents

**Table S1:** Participant Characteristics at Time of First Infection

**Figure S1:** Number of cases by week for all included infections by infection number.

**Table S2:** All included infections and confirmation method by infection first or second infection.

**Table S3:** Infection severity of first and second infections by vaccination and serological response

Table S1: Participant Characteristics at Time of First Infection. Averages are presented with standard deviations, while counts are presented with percentages.

|  | n = 739 |
| --- | --- |
| Age (years) | 46.2 (11.5) |
| Sex |  |
| Female | 599 (81.1%) |
| Initial vaccine series |  |
| Pfizer | 573 (77.5%) |
| Not vaccinated | 106 (14.3%) |
| Moderna | 43 (5.8%) |
| Mixed | 7 (0.9%) |
| Johnson & Johnson | 6 (0.8%) |
| AstraZeneca | 4 (0.5%) |
| Race-ethnicity |  |
| Non-Hispanic white | 603 (81.6%) |
| Non-Hispanic non-white | 65 (8.8%) |
| Hispanic white | 71 (9.6%) |
| Study |  |
| IASO | 682 (92.3%) |
| HICS | 57 (7.7%) |


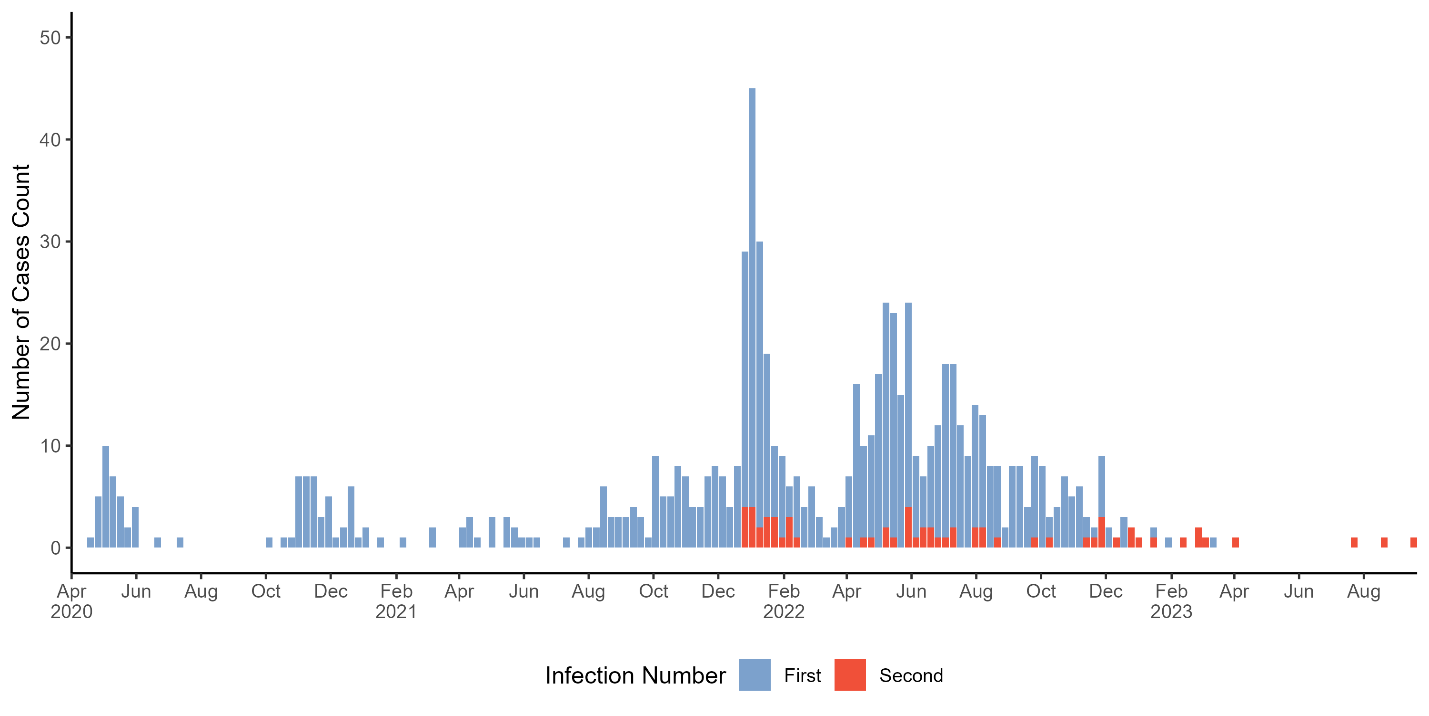


Figure S1: Number of cases by week for all included infections by first or second infection. Blue are first infections, and red are second infections.

Table S2: All included infections and confirmation method by infection first or second infection.

|  | First Infections | Second Infections |
| --- | --- | --- |
| RT-PCR | 546 (78.0%) | 53 (81.5%) |
| Rapid or Reported | 149 (21.3%) | 12 (18.5%) |
| Spike serology | 5 (0.7%) | 0 |
| Totals | 700 | 65 |

Table S3: Infection severity of first and second infections by vaccination and serological response where mild cases are those that did not require medical attention or have reports of respiratory discomfort. P-values are calculated with χ^2^.

| Infections with Mild Symptom Presentation | | |
| --- | --- | --- |
| First SARS-CoV-2 Infections | | **P Value** |
| Vaccinated | 97.60% | <.0001 |
| Unvaccinated | 84.80% |  |
|  |  |  |
| Seroconverted | 96.40% | 0.2838 |
| Did Not Seroconvert | 98.70% |  |
| SARS-CoV-2 Reinfections | | **P Value** |
| Vaccinated | 91.30% | 0.7657 |
| Unvaccinated | 88.90% |  |
|  |  |  |
| >= 4-Fold Rise | 89.70% | 0.4079 |
| < 4-Fold Rise | 100% |  |
